# VCP mutations and parkinsonism: An emerging link

**DOI:** 10.1016/j.prdoa.2023.100230

**Published:** 2023-12-01

**Authors:** Jumana T. Alshaikh, Ashley Paul, Emile Moukheiber, Sonja W. Scholz, Alexander Pantelyat

**Affiliations:** a Department of Neurology, University of Utah School of Medicine, United States; b Department of Neurology, Johns Hopkins School of Medicine, United States; c Neurodegenerative Diseases Research Unit, National Institute of Neurological Diseases and Stroke, United States

**Keywords:** Parkinsonism, VCP mutations

Valosin-containing protein (VCP) is an enzyme in the ATPase family involved in protein homeostasis. VCP mutations are associated with various degenerative conditions, including frontotemporal dementia (FTD), inclusion body myopathy (IBM), Paget’s disease, amyotrophic lateral sclerosis, and Charcot-Marie-Tooth disease. Furthermore, parkinsonism has been reported in several VCP mutation carriers (Fig. 1A). Here, we present two novel cases with VCP-associated parkinsonism. The first patient had a R191G mutation which has only been reported in association with parkinsonism in a single family [1]. The second patient carried a pathogenic R155C mutation, which was reported in one case only in association with parkinsonism [2].

**Fig. 1:**
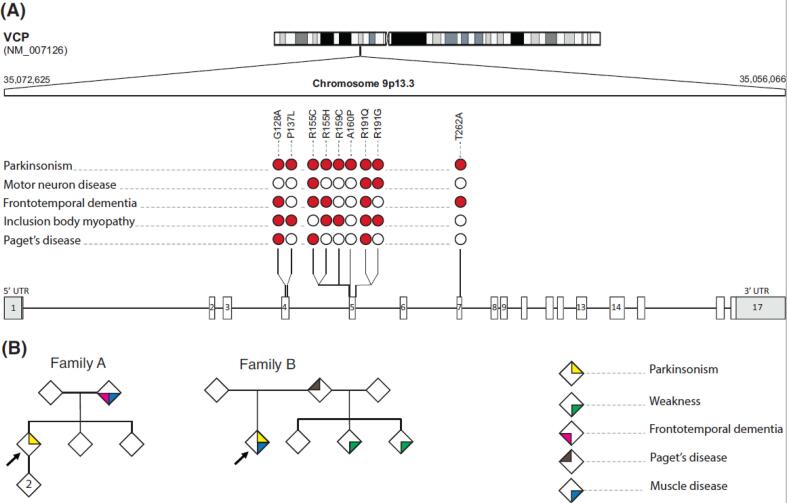
Mutations in VCP-associated parkinsonism patients. (A) This graphical representation illustrates the clinical heterogeneity of VCP-associated parkinsonism. An ideogram of chromosome 9 is shown at the top of the figure with an enlarged view of the VCP gene location and the observed VCP mutations underneath. Red circles denote the presence of a specific phenotype. All mutations are depicted relative to the canonical sequence NM_007126 (hg38). (B) depicts the pedigrees of the two parkinsonism cases presented in this article. In family A, a pathogenic, segregating R191G mutation was found, while affected members in family B carried the pathogenic R155C mutation. (For interpretation of the references to colour in this figure legend, the reader is referred to the web version of this article.)

Our first case is a 53-year-old male who presented for evaluation of tremor. His first neurological symptom was weakness in his lower extremities due to IBM. Six years later, he developed a left hand resting and action tremor. On review of systems, he reported anxiety, depression, and insomnia. He reported no hyposmia, dream enactment, constipation, or orthostatic dizziness. His mother had FTD with onset in her mid-fifties, IBM, and a tremor. His exam was notable for weakness in his deltoids, biceps, abductor pollicis brevis, iliopsoas, quadriceps, toe flexors and extensors, and parkinsonism manifesting with neck rigidity, decrementing bradykinesia, left hand resting tremor, and postural instability with a total MDS-UPDRS motor score of 34/132. He had no abnormalities on oculomotor or vestibular testing. On neuropsychological testing, he had mild impairment on a measure of letter fluency, but overall his performance was normal. On genetic testing, we found a pathogenic mutation in the VCP gene, c.572G > A (p.Arg191Gln). He was started on carbidopa/levodopa for his parkinsonism but reported no benefit with levodopa doses of up to 1,200 mg/day. He developed respiratory muscle weakness and died of respiratory failure at age 56.

Our second case is a 40-year-old male who presented with two years of adult-onset, progressive, asymmetric, hip flexion, elbow flexion, and scapular weakness. He had an extensive patrilineal family history of muscular dystrophy of unknown etiology with manifestations including weakness and possible visceral complications suggesting an autosomal dominant inheritance pattern (Fig. 1B). His father was diagnosed with muscular dystrophy and Paget’s disease. He died in his late 40 s due to cardiac disease and hepatic disease. One half-brother was wheelchair-bound by his late 40 s; the other half-brother required a walker by his early 50 s and was diagnosed with fatty liver disease. One year after symptom onset, our patient developed asymmetric tremor-predominant parkinsonism with a robust response to levodopa (1,750 mg/day). His creatine kinase was marginally elevated at 374 U/L. Electromyography, muscle biopsy, brain and cervical spine MRI were unrevealing for the underlying etiology. A limb-girdle muscular dystrophy panel (Athena) showed a heterozygous SGCA mutation (349C > T, PARG117TRP). He also carried a variant of unknown significance in PLEC (c.8643 T > C), which he shared with his older, affected half-brother. He was ultimately found to be heterozygous for the pathogenic c.463C > T (p.Arg155Cys) VCP mutation, confirming the diagnosis of IBM associated with Paget’s disease of bone and/or FTD (IBMPFD) with parkinsonism.

While VCP mutations resulting in parkinsonism are rare—in one study of 231 patients with 15 different VCP mutations, 3.8 % of patients had parkinsonism [3]—it is an important emerging link that should be considered in patients with both neuromuscular disorders and parkinsonism phenotypes. VCP is an enzyme involved in protein degradation. In one study there was a reduction in VCP expression in PD, which may play a role in the accumulation of misfolded proteins [4]. Furthermore, on immunohistochemical examination of the substantia nigra of PD patients, VCP was found within Lewy bodies and neurites, and Marinesco bodies; therefore, VCP mutations may be implicated in the aggregation of these abnormal inclusions [5].

While codon 155 is a mutation hotspot, R191G and R155C mutations associated with parkinsonism are extremely rare. Unlike the prior case report of a patient with an R155C mutation who presented with parkinsonism followed by IBMPFD [2], our patient had an initial presentation of Paget’s disease of bone followed by parkinsonism one year later. This observation highlights the phenotypic heterogeneity of VCP-associated parkinsonism, ranging from isolated parkinsonism to mixed presentations (Fig. 1A). The existing literature suggests that VCP-parkinsonism has a robust levodopa responsiveness [3]. However, our patient with the R191G mutation was not levodopa-responsive; the previously published case did not comment on levodopa response [1]. Future studies should look at whether there is a correlation between genotype, phenotype and levodopa responsiveness, which can be useful in discussions of expected treatment response and quality of life.

## Declaration of competing interest

The authors declare that they have no known competing financial interests or personal relationships that could have appeared to influence the work reported in this paper.
